# Cataloging the Genetic Response: Unveiling Drought-Responsive Gene Expression in Oil Tea Camellia (*Camellia oleifera* Abel.) through Transcriptomics

**DOI:** 10.3390/life14080989

**Published:** 2024-08-08

**Authors:** Zhen Zhang, Yanming Xu, Caixia Liu, Longsheng Chen, Ying Zhang, Zhilong He, Rui Wang, Chengfeng Xun, Yushen Ma, Xiaokang Yuan, Xiangnan Wang, Yongzhong Chen, Xiaohu Yang

**Affiliations:** 1 Hunan Academy of Forestry, Changsha 410000, China; hfazz@hnlky.cn (Z.Z.); xuyanming@hnlky.cn (Y.X.); lcaixia1989@hnlky.cn (C.L.); clongsheng@163.com (L.C.); zhangying@hnlky.cn (Y.Z.); hezhilong2000@hnlky.cn (Z.H.); wangrui2019@hnlky.cn (R.W.); xunchengfeng24@163.com (C.X.); mys9204@163.com (Y.M.); wxn@hnlky.cn (X.W.); 2 National Engineering Research Center for Oil Tea Camellia, Changsha 410000, China; 3 Hunan Key Laboratory of Meteorological Disaster Prevention and Reduction, Hunan Research Institute of Meteorological Sciences, Changsha 410000, China; yxknuist@126.com

**Keywords:** *Camellia oleifera*, drought stress, transcriptome sequence, DEGs

## Abstract

Drought stress is a critical environmental factor that significantly impacts plant growth and productivity. However, the transcriptome analysis of differentially expressed genes in response to drought stress in *Camellia oleifera* Abel. is still unclear. This study analyzed the transcriptome sequencing data of *C. oleifera* under drought treatments. A total of 20,674 differentially expressed genes (DEGs) were identified under drought stress, with the number of DEGs increasing with the duration of drought. Specifically, 11,793 and 18,046 DEGs were detected after 8 and 15 days of drought treatment, respectively, including numerous upregulated and downregulated genes. Gene Ontology (GO) enrichment analysis showed that the DEGs were primarily involved in various biological processes. Kyoto Encyclopedia of Genes and Genomes (KEGG) pathway enrichment analysis revealed that carbon metabolism, glyoxylate and dicarboxylate metabolism, proteasome, glycine, serine, and threonine metabolism were the main affected pathways. Among the DEGs, 376 protein kinases, 42 proteases, 168 transcription factor (TF) genes, and 152 other potential functional genes were identified, which may play significant roles in the drought response of *C. oleifera*. The expression of relevant functional genes was further validated using quantitative real-time PCR (qRT-PCR). These findings contribute to the comprehension of drought tolerance mechanisms in *C. oleifera* and bolster the identification of drought-resistant genes for molecular breeding purposes.

## 1. Introduction

Drought stress is one of the most significant environmental factors affecting plant growth and productivity [1]. Abiotic stressors frequently encountered in nature can significantly impact plant life, disrupting their growth and development, hindering photosynthetic efficiency, impairing water and nutrient uptake, and ultimately affecting yield [2,3,4]. Understanding the molecular mechanisms underlying plant responses to drought is crucial for developing strategies to enhance drought tolerance. Throughout the course of evolution, plants have evolved sophisticated physiological, biochemical, molecular, and morphological adaptations to effectively cope with drought stress [5,6]. Morphological strategies, such as reducing leaf size, regulating stomatal closure, adjusting flowering time, and stimulating root growth, contribute to enhanced drought tolerance [7]. Physiological and biochemical mechanisms involve the establishment of antioxidant defense systems [8], repair of cell membrane damage, and regulation of hormone levels [9]. For example, under drought conditions, the accumulation of reactive oxygen species (ROS) and malondialdehyde (MDA) can induce oxidative stress and membrane injury, impacting plant growth and survival. In response, plants activate antioxidant enzymes such as superoxide dismutase (SOD), catalase (CAT), peroxidase (POD), ascorbate peroxidase (APX), as well as osmotic regulators like soluble sugars (SS) and soluble proteins (SP) to mitigate ROS levels, restore membrane integrity, and bolster drought resistance.

At the molecular level, researchers have utilized high-throughput sequencing technologies to investigate plant responses to drought stress. Transcriptomics and bioinformatics are crucial for understanding gene expression and functional genomics, as they enable a comprehensive analysis of gene activity and interactions under various conditions [10,11]. These techniques provide valuable insights into the molecular mechanisms underlying complex traits, including stress responses [12,13]. Studies have revealed that drought stress triggers the expression of specific functional and regulatory genes and proteins in plants. Some of these proteins, such as antioxidant proteins and various proteases, directly contribute to drought resistance [14,15]. Others, such as protein kinases, transcription factors, and hormone-related genes, modulate drought response pathways, thereby enhancing the plant’s ability to withstand drought stress [16,17]. A plant’s response to drought stress is a complex system involving interconnected components, highlighting the need for further research to deepen our understanding of this phenomenon and to refine strategies for enhancing drought resilience. Recent studies have explored transcriptomic analyses and drought stress responses in various plants such as maize (*Zea mays*) and soybeans (*Glycine max*) [18,19]. However, it is important to provide new insights specific to *C. oleifera* and enhance the understanding of drought resistance genes in this economically valuable species.

Oil tea camellia (*Camellia oleifera* Abel.), a valuable woody plant prized for its premium edible oil, is extensively cultivated. Despite its economic significance, the precise molecular mechanisms underlying *C. oleifera*’s response to drought stress remain elusive. Belonging to the *Theaceae* family, *C. oleifera* is a perennial small shrub or tree of considerable importance as a woody oil crop in China, boasting both economic and ecological benefits [15,20]. Renowned for its high content of unsaturated fatty acids, camellia seed oil is esteemed for its nutritional value [21]. This species predominantly thrives in the mountainous and hilly regions of southern China [22]. Drought poses a significant challenge to the growth and yield of *C. oleifera*, necessitating urgent research into drought resistance [23]. Several studies have been conducted on drought in camellia plants. Strategies such as organic mulching have shown promise in enhancing *C. oleifera*’s drought resilience by modulating soil water potential, temperature, and the expression of genes associated with stress responses [24]. Additionally, the endophyte Streptomyces albidoflavus OsiLf-2 has been identified as beneficial for *C. oleifera*, mitigating drought stress by altering the rhizosphere microbial community, reducing root water loss, and synthesizing osmoprotectants and antioxidants [25]. Through mechanisms involving increased levels of phytohormones, enzymatic activities, and osmolytes, *C. oleifera* exhibits enhanced antioxidant capacity and water regulation under drought conditions [26,27]. However, these studies mainly focus on the physiological characteristics and cultivation measures of *C. oleifera’s* drought resistance, and only few studies have analyzed the differential genes associated with drought resistance in *C. oleifera* through transcriptome sequencing. The result is that the understanding of the molecular basis of drought resistance in *C. oleifera* remains limited [28,29,30,31,32], highlighting the need for comprehensive research in this area. In addition, there are currently no drought resistance studies on ‘Xianglin 210’ *C. oleifera*—the main variety in the development of China’s oil tea industry with the largest planting area—based on transcriptome sequencing.

In this study, our primary goal is to investigate the differentially expressed genes (DEGs) in three-year-old ‘Xianglin 210’ *C. oleifera* grafted container seedlings exposed to simulated drought conditions in pot experiments. Our research objectives were as follows: (1) to utilize transcriptome sequencing to identify genes linked to drought resistance in ‘Xianglin 210’ *C. oleifera*; and (2) to provide valuable insights that can guide breeding initiatives and cultivation techniques, enhance the production of ‘Xianglin 210’ *C. oleifera*, and foster the development of drought-resistant varieties of *C. oleifera*.

## 2. Materials and Methods

### 2.1. Study Site

**Figure 1:**
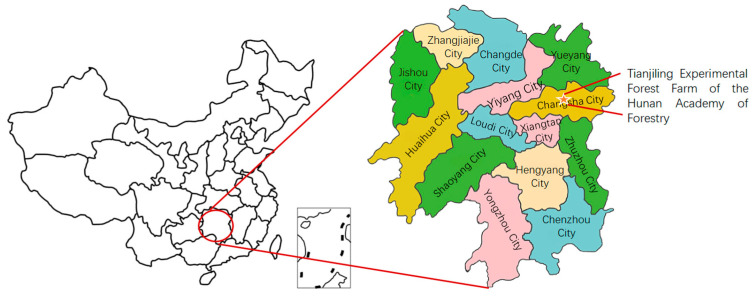
Study site.

The study site was located at the Tianjiling Experimental Forest Farm of the Hunan Academy of Forestry (113°01′ E, 28°06′ N) with an elevation ranging from 80 to 100 m (Figure 1). 

### 2.2. Plant Materials, Drought Treatment, and Experimental Design

To simulate drought conditions in the actual production of *C. oleifera*, the experiment was conducted from 31 July to 15 August 2023, a period when *C. oleifera is* most susceptible to drought due to high physiological water demands and concurrent high temperatures and scarce rainfall.

**Figure 2:**
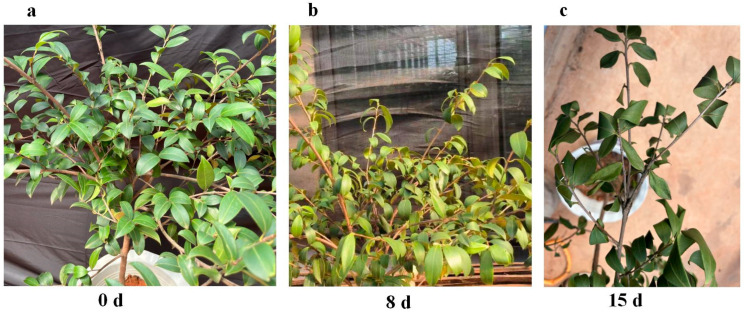
Phenotypic changes of *C. oleifera* under drought stress. (**a**) 0 d; (**b**) 8 d; and (**c**) 15 d.

We conducted a pot experiment using 60 container-grown seedlings of ‘Xianglin 210’ *C. oleifera*, each aged three years, as the experimental specimens. The selection of three-year-old grafted seedlings aligns with prevailing practices in *C. oleifera* afforestation. Three specific time points (0-day, 8-day, and 15-day) were chosen based on previous experimental studies [27,33,34], which indicated significant physiological changes in response to drought stress within a 15-day period. The experiment followed a completely randomized design (CRD). The 60 seedlings were divided into three groups, with each group consisting of 20 seedlings, to provide three replications for measurements at three specific time periods. On 31 July 2023, all seedlings were thoroughly watered and then placed in the field under a plastic roof cover to prevent natural rainfall. All other environmental factors were kept constant, with the exception of rainfall. Leaf samples were collected from grafted seedlings at each time point: 0 days, 8 days, and 15 days after drought stress (Figure 2). For each time point, samples were taken from three groups as three replicates. For each replicate, the top, middle, and bottom leaves from 20 seedlings were collected and combined as mixed samples. Figure 2 illustrates the drought status at these specific time periods. After rinsing with deionized water, the samples were frozen in liquid nitrogen and stored at −70 °C for further analysis, including transcriptome sequencing and the measurement of relative leaf water content (RWC).

### 2.3. Measurements and Analysis

#### 2.3.1. Soil Volumetric Moisture Content Measurement

Soil volumetric moisture content was assessed using a hand-held meteorological environment detector (LD-QX008, Shandong Lainde Intelligent Technology Institute, Shandong, Wei Fang, China).

#### 2.3.2. RWC Measurement

RWC was determined using the following equation: $\mathrm{RWC}=((m_{1}-m_{2})/{(m}_{1}-m_{3}))\times 100\%.$ m_1_ is the mass of the weighing bottle and the sample, m_2_ is the mass of the weighing bottle and the sample after drying, and m_3_ is the mass of the weighing bottle.

#### 2.3.3. Antioxidant Enzyme Activities Measurement

The activity of POD was measured using a peroxidase assay kit (JC0102-S, Nanjing Jice Biotechnology Institute, Nanjing, China) according to the manufacturer’s instructions. CAT activity was evaluated using a catalase assay kit (JC0103-S, Nanjing Jice Biotechnology Institute, Nanjing, China).

#### 2.3.4. RNA Isolation and Qualification

RNA was extracted using the TRIzol method (Invitrogen, Carlsbad, CA, USA) and subsequently treated with RNase-free DNase I (Takara, Kusatsu, Japan). The integrity and potential contamination of the RNA were assessed by running samples on 1% agarose gels. Quantification of RNA was performed using an Agilent 2100 Bioanalyzer (Agilent Technologies, Santa Clara, CA, USA), while the quality and integrity were further evaluated with a NanoDrop spectrophotometer (Thermo Scientific, Wilmington, DE, USA).

#### 2.3.5. Library Preparation for Transcriptome Sequencing

Sequencing libraries were generated using NEBNext^®^ Ultra™ RNA Library Prep Kit for Illumina^®^ (NEB, Ipswich, MA, USA). Index codes were incorporated to assign sequences to each sample. To select cDNA fragments with a preferential length of 200–250 bp, library fragments were purified using the AMPure XP system (Beckman Coulter, Beverly, CA, USA). Following this, PCR products were purified using the same AMPure XP system. The quality of the libraries was assessed with the Agilent Bioanalyzer 2100 system. Finally, the library preparations were sequenced on an Illumina Novaseq 6000 platform by the Beijing Allwegene Technology Company Limited (Beijing, China).

#### 2.3.6. Cleaning and Mapping of Sequenced Reads

Raw data (raw reads) in FASTQ format were initially processed using in-house Perl scripts. Clean data (clean reads) were generated by removing reads containing adapters, reads with poly-N sequences, and low-quality reads from the raw data. Additionally, Q20, Q30, GC content, and sequence duplication level of the clean data were calculated.

#### 2.3.7. Differential Expression Analysis

Differential expression analysis of two conditions/groups was performed using the DESeq R package (1.10.1). The resulting *p* values were adjusted using Benjamini and Hochberg’s approach to control the false discovery rate. Genes with an adjusted *p*-value < 0.05, as determined by DESeq, were considered differentially expressed.

#### 2.3.8. GO and KEGG Pathway Enrichment Analysis

Gene Ontology (GO) enrichment analysis of the DEGs was conducted using the GO seq R package, which employs the Wallenius non-central hypergeometric distribution. Statistical enrichment of DEGs in Kyoto Encyclopedia of Genes and Genomes (KEGG) pathways was assessed using KOBAS.

#### 2.3.9. Quantitative Real-Time PCR Analysis

For qRT-PCR validation, 7 DEGs were selected, and Tubulin was identified as the internal reference gene. Gene expression levels were relatively quantified using the 2−∆∆Ct method.

### 2.4. Statistical Analysis

Analysis of variance (ANOVA) was used to statistically assess the net effects of drought treatment on gene expression, including all dependent variables. All statistical analyses were conducted using SPSS 16.0 (IBM SPSS Inc., Armonk, NY, USA). Multiple comparisons used the least significant difference test (LSD).

## 3. Results

### 3.1. Phenotypic Observation and Physiological Analysis

After exposure to drought conditions, the leaves of *C. oleifera* exhibited clear symptoms of wilting and curling (Figure 2). Soil volumetric moisture content was 34.57% at day 0, declines significantly to 13.95% by day 8, and further decreased to 9.34% by day 15. Table 1 details the physiological responses of *C. oleifera* to drought stress over 15 days, specifically focusing on POD, CAT, and RWC. Initially, at day 0, POD activity is 32.31 U g^−1^ min^−1^ FM, significantly increases to 35.00 U g^−1^ min^−1^ FM by day 8, and further increases to 40.65 U g^−1^ min^−1^ FM by day 15. CAT activity, which begins at 26.09 U g^−1^ min^−1^ FM on day 0, shows a gradual increase to 26.19 U g^−1^ min^−1^ FM by day 8, and rises sharply to 66.20 U g^−1^ min^−1^ FM by day 15. Both POD and CAT activities peaked at 15 days, showing significant increases compared to day 0 (Table 1), indicating an adaptive mechanism to combat increasing oxidative damage. Concurrently, RWC starts at a high of 60.90%, indicative of healthy hydration levels, but decreases to 55.16% by day 8, and drops significantly to 43.44% by day 15, highlighting the severe water deficit experienced by the leaves under prolonged drought conditions.

### 3.2. Transcriptome Sequencing and Assembly

**Figure 3:**
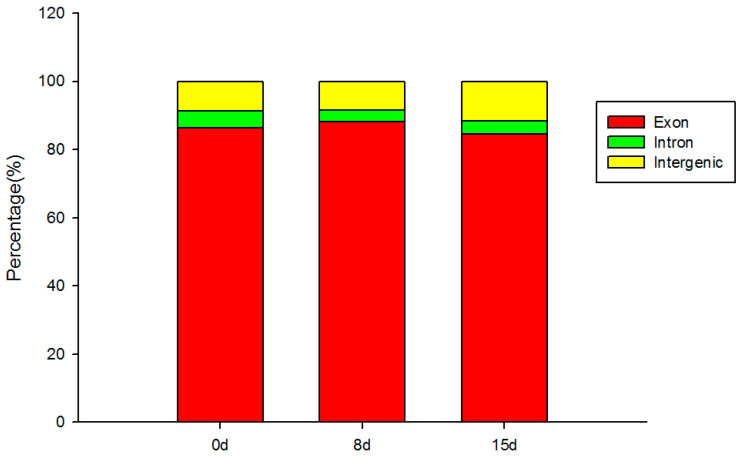
Distribution of reads in different regions of *C. oleifera* genome.

In this study, we conducted transcriptome analysis to explore the changes in gene expression in *C. oleifera* under drought stress using leaf tissue samples. We constructed a total of nine cDNA libraries, which collectively generated 55.23 Gb of raw sequencing data. Each library produced between 38,356,506 and 44,590,766 raw reads. Following stringent quality control measures to remove low-quality reads, each library retained between 36,844,334 and 42,556,954 clean reads, representing over 95.14% of the total reads (Supplementary Table S1). The high sequencing quality was evident from Q30 values, ranging from 96.22% to 96.45%, indicating minimal error rates and ensuring the reliability of the dataset for subsequent analyses (Supplementary Table S1). The subsequent alignment of clean reads from each sample to the reference genome revealed successful mapping rates ranging from 94.80% to 95.78% per library. The GC content of mapped reads ranged from 44.41% to 45.85%, consistent with typical plant genomes (Supplementary Table S1). Notably, the majority of mapped reads were localized to exon regions, demonstrating the specificity of our sequencing approach in capturing transcribed regions of the genome (Figure 3).

### 3.3. Identification and Analysis of DEGs

Comparing the time points 0 d vs. 8 d, we identified 11,793 DEGs, with 5472 genes upregulated and 6321 genes downregulated. Similarly, in the comparison of 0 d vs. 15 d, we detected 18,046 DEGs, comprising 8534 upregulated and 9512 downregulated genes.

**Figure 4:**
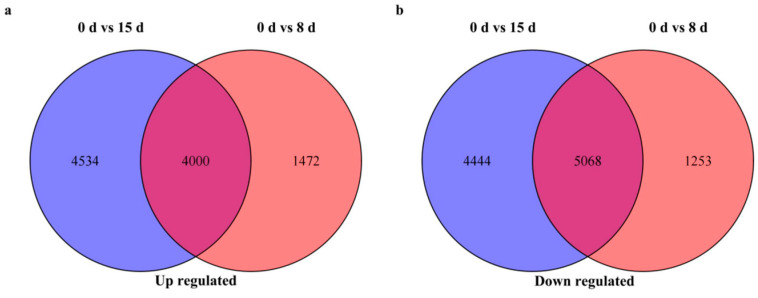
Venn diagram analysis of DEGs at different time points. (**a**) Upregulated DEGs and (**b**) downregulated DEGs.

After removing duplicates, a total of 20,674 unique DEGs were identified in *C. oleifera* in response to drought stress. A Venn diagram analysis among the 0 d vs. 8 d and 0 d vs. 15 d comparisons and their overlap revealed 9165 DEGs. Among these, 4000 genes were upregulated, while 5068 genes were downregulated (Figure 4).

### 3.4. GO and KEGG Enrichment Analysis of DEGs

Prolonged drought stress elicits significant changes in the molecular pathways and functional categories of *C. oleifera*, as evidenced by the comprehensive GO and KEGG enrichment analyses of DEGs.

In the comparison of 0 d vs. 8 d, DEGs were enriched in a total of 4147 GO terms, distributed across biological process (2147), molecular function (1193), and cellular component (537) categories. Similarly, in the comparison of 0 d vs. 15 d, DEGs were enriched in 4396 GO terms, comprising biological process (2533), molecular function (1292), and cellular component (571) categories.

Further investigation of the top 20 enriched GO terms revealed consistent themes across both time points. Key terms such as biological process, cellular process, photosystem II oxygen-evolving complex, thylakoid membrane, catabolic process, oxidoreductase complex, photosynthetic membrane, thylakoid, and photosystem were prominently enriched among the DEGs in both 0 d vs. 8 d and 0 d vs. 15 d comparisons (Supplementary Table S2). These findings highlight the adaptive strategies of *C. oleifera* under drought conditions, focusing on energy production, stress response, and metabolic regulation.

In addition to the GO analysis, the KEGG pathway enrichment analysis identified significant pathways associated with drought response. In 0 d vs. 8 d, 6057 DEGs were enriched in 122 pathways, while in 0 d vs. 15 d, 7896 DEGs were enriched in 106 pathways. Commonly enriched pathways included carbon metabolism, glyoxylate and dicarboxylate metabolism, proteasome, glycine, serine, and threonine metabolism. The top 20 significant pathways in both comparisons provided further insights into the molecular mechanisms involved in *C. oleifera*’s adaptation to drought stress (Table 2).

**Figure 5:**
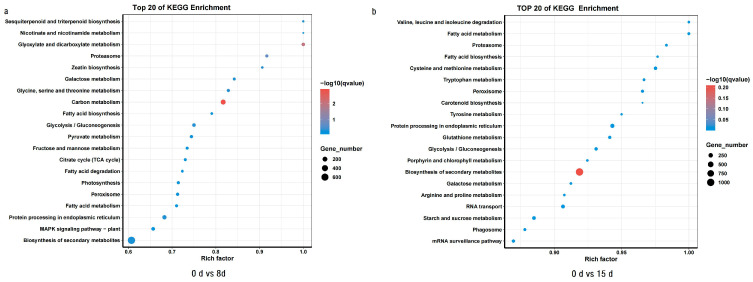
Top 20 KEGG enrichment pathways under drought treatment. Rich factor represents the ratio of the number of DEGs in the pathway.(**a**) Top 20 KEGG enichment between 0 d vs 8 d and (**b**) Top 20 KEGG enichment between 0 d vs 15 d.

Specifically, pathways such as glycine, serine, and threonine metabolism, photosynthesis, and the MAPK-signaling pathway plant were enriched in the 0 d vs. 8 d comparison, emphasizing their roles in stress response and signaling (Figure 5). Meanwhile, pathways related to cysteine and methionine metabolism, porphyrin, and chlorophyll metabolism were significantly enriched in the 0 d vs. 15 d comparison, highlighting the plant’s metabolic adjustments and photo-protective responses under prolonged drought stress.

Protein kinases and proteases are crucial for plants to effectively withstand drought stress. In this study, we identified 376 protein kinase-related genes (Supplementary Table S3), encompassing mitogen-activated protein kinase (MAPK), calcium-dependent protein kinases (CDPKs/CPKs), receptor-like kinases (RLKs), and serine/threonine–protein kinases, with the latter being the most abundant. Additionally, 42 protease-related genes were identified (Supplementary Table S3), including serine proteases, cysteine proteases, aspartic proteases, and metalloproteases. These genes exhibited significant upregulation or downregulation under prolonged drought stress, suggesting critical roles for these protein kinase and protease families in *C. oleifera*’s response to drought stress through regulatory mechanisms.

By regulating the expression of specific genes, transcription factors (TFs) can significantly influence the physiological and biochemical processes of plants, thereby enhancing their ability to adapt to or resist drought stress. In our study, we identified a total of 168 transcription factors, predominantly belonging to families such as MYB, WRKY, ERF, NAC, and bHLH (Supplementary Table S4). Among these families, MYB exhibited the highest abundance. Furthermore, we observed that under prolonged drought stress conditions, the expression of most transcription factor genes gradually increased.

Drought stress response is a complex system. We also identified 152 other important DEGs (Supplementary Table S5), including functional genes related to hormones, antioxidant enzymes, F-box proteins, and zinc finger proteins. The expression patterns of these DEGs vary, with the majority showing upregulation. This suggests that these DEGs may act as both positive and negative regulators in *C. oleifera*’s adaptation to drought stress.

### 3.5. Verification of Selected DEGs

The genes selected for verification included three protein kinases, one protease, and three transcription factors (Supplementary Table S6). Tubulin was used as the internal reference gene. The qRT-PCR analysis showed that the expressions of these seven genes were upregulated under drought conditions (Table 3). Generally, the expression patterns of these genes were consistent with the results of transcriptome sequencing, confirming the reliability of the transcriptome sequencing results.

## 4. Discussion

Drought stress is a major environmental factor that adversely affects plant growth and productivity, posing significant challenges to agricultural systems worldwide. During drought conditions, the primary factor leading to leaf wilting in plants is the loss of turgor pressure, which is primarily caused by water deficiency and osmotic stress [35]. After exposure to drought conditions, the leaves of *C. oleifera* exhibited clear symptoms of wilting and curling, indicative of the plant’s physiological response to water scarcity. These symptoms are primarily associated with a loss of turgor pressure within the leaf cells, which occurs when water availability is limited, leading to osmotic stress [36]. As the drought persists, the plant’s ability to maintain cellular water balance is compromised, resulting in visible morphological changes such as leaf wilting and curling [37]. This is consistent with research findings on *Arachis hypogaea* [33] and *Carya illinoinensis* [34], indicating that drought causes physiological damage to *C. oleifera*. Our findings align with the hypothesis that oxidative stress may compromise cellular integrity and physiological functions in *C. oleifera* under water deficit conditions.

The significant decrease in soil volumetric moisture content after 15 days of drought treatment (Table 1) underscores the severity of water deficit experienced by the plants during the experimental period. This reduction in soil moisture content emphasizes the plant’s diminishing ability to absorb water, directly impacting its water status and leading to turgor loss in the leaves [38]. Similar decreases in soil moisture content have been reported in other drought studies, highlighting the robustness of our experimental setup [39,40]. Antioxidant enzymes such as POD and CAT are crucial components of the plant defense mechanism against oxidative stress [41,42,43]. Our results indicate that both POD and CAT (Table 1) activities increased significantly after 15 days of drought treatment compared to day 0. This suggests an adaptive response of *C. oleifera* to mitigate oxidative damage by enhancing enzymatic antioxidant defense systems. The gradual decrease in RWC observed over the course of the drought treatment (Table 1) is indicative of progressive water loss and dehydration stress in *C. oleifera* leaves. Similar findings have been reported in other plant species subjected to drought conditions, highlighting the universality of RWC as a physiological indicator of water status [44,45].

Understanding how plants respond at the molecular level to drought stress is crucial for developing strategies to enhance their resilience. This comprehensive transcriptome analysis provides valuable insights into the molecular responses of *C. oleifera* to drought stress. The identification of 20,674 DEGs under drought conditions underscores the complexity of the plant’s response mechanisms. Our findings align with previous studies that have utilized transcriptome analysis to decipher plant responses to abiotic stressors [46,47]. Research on other plant species, such as *Arabidopsis thaliana* and rice (*Oryza sativa*), has elucidated key genes and pathways involved in stress perception, signal transduction, and physiological adaptations [48,49]. Drought stress is a major environmental challenge affecting plant growth and productivity, necessitating adaptive responses at the molecular level. In this study, we employed transcriptome analysis to investigate the differential gene expression in *C. oleifera* under varying durations of drought stress. The comparisons between different time points (0 d vs. 8 d and 0 d vs. 15 d) revealed substantial changes in the transcriptomic landscape, highlighting the plant’s dynamic response to prolonged water-deficit conditions. This progressive increase in the number of DEGs over time underscores the complexity and depth of the plant’s molecular adjustments as drought stress prolongs. Notably, at both time points, the number of downregulated DEGs exceeded that of upregulated genes, suggesting a prioritization of gene suppression mechanisms to conserve resources and enhance stress tolerance [50,51]. A Venn diagram analysis further elucidated the overlap and distinctiveness of DEGs between the two comparisons. These shared DEGs likely represent core components of *C. oleifera*’s drought stress response, potentially influencing essential pathways such as stress signaling, hormone regulation, and metabolic adjustments through both positive and negative mechanisms [52,53]. The upregulation of specific genes, including those encoding antioxidant enzymes like peroxidases and catalases, suggests an active defense mechanism against oxidative stress induced by drought [8,54]. Concurrently, the observed downregulation of genes associated with growth and development may reflect the plant’s adaptive strategy to prioritize survival under adverse environmental conditions [55,56].

Drought stress poses a significant challenge to plant survival and productivity, necessitating intricate molecular adaptations in species like *C. oleifera*. This study employed comprehensive transcriptomic analyses to explore the changes in gene expression patterns and associated pathways in response to prolonged drought stress. The GO and KEGG enrichment analyses of DEGs provided insights into the molecular mechanisms underlying *C. oleifera*’s response to water-deficit conditions. In both comparisons of 0 d vs. 8 d and 0 d vs. 15 d, a substantial number of DEGs were identified and enriched in diverse GO categories. The predominance of biological process categories in both comparisons highlights the plant’s prioritization of physiological adjustments and metabolic reprogramming in response to drought stress. Further analysis of the top 20 enriched GO terms revealed consistent themes across both time points. These findings highlight the adaptive strategies of *C. oleifera* to sustain essential cellular functions under adverse environmental conditions, reflecting the plant’s resilience to drought-induced challenges. The KEGG pathway enrichment analysis identified critical pathways involved in *C. oleifera*’s drought response. Key pathways such as carbon metabolism, glyoxylate and dicarboxylate metabolism, proteasome, and amino acid metabolism including glycine, serine, and threonine pathways were commonly enriched in both comparisons (Figure 5). These pathways are essential for maintaining cellular homeostasis, energy production, and stress signaling under drought conditions [48,57]. Specifically, in the 0 d vs. 8 d comparison, pathways associated with photosynthesis and MAPK-signaling pathway plant were enriched, highlighting their roles in energy capture and stress perception (Figure 5). Conversely, in the 0 d vs. 15 d comparison, pathways related to cysteine and methionine metabolism, and porphyrin and chlorophyll metabolism were significantly enriched, indicating adjustments in metabolic pathways crucial for photo-protection and stress tolerance [53].

Protein kinases and proteases play crucial roles in enabling plants to cope with environmental stresses, particularly drought, by regulating signaling pathways and protein turnover mechanisms. In this study, we identified a comprehensive set of protein kinase and protease genes in *C. oleifera* that respond dynamically to drought stress. Among these protein kinases, serine/threonine–protein kinases were the most abundant, reflecting their central role in mediating stress responses in plants [57,58]. These proteases are essential for protein degradation and turnover, processes critical for adapting to stress conditions and maintaining cellular homeostasis [59,60]. Under prolonged drought stress, many of these identified genes showed significant changes in expression levels, indicating their active involvement in *C. oleifera*’s response to water deficit. Both upregulation and downregulation of these genes suggest their roles in regulating adaptive processes through positive or negative regulatory mechanisms [61,62]. The findings underscore the complexity of the molecular mechanisms underlying *C. oleifera*’s adaptation to drought stress, where protein kinases and proteases act as key players in signaling cascades and protein metabolism. Future studies focusing on specific kinase–protease interactions and their downstream targets will provide deeper insights into the regulatory networks governing plant responses to environmental challenges.

By modulating the expression of specific genes, transcription factors (TFs) play a crucial role in regulating various physiological and biochemical processes in plants, thereby enhancing their ability to adapt to environmental stresses such as drought. In our investigation of *C. oleifera*, we identified multiple transcription factors. The observed upregulation of transcription factor genes under prolonged drought stress conditions in *C. oleifera* suggests their potential involvement in the plant’s adaptive responses. MYB transcription factors, for instance, have been implicated in regulating pathways related to stress responses, secondary metabolism, and developmental processes [63,64]. WRKY TFs are known to play roles in both biotic and abiotic stress responses by regulating the expression of defense-related genes [65]. ERF TFs, on the other hand, are involved in ethylene-mediated signaling pathways that activate stress-responsive genes [66]. Overexpression of bHLH can improve plant response to drought stress by promoting photosynthesis, accumulating Pro, enhancing antioxidant enzyme activity, and inhibiting ROS damage [67,68,69,70]. NAC genes can enhance drought tolerance of Triticum aestivum by increasing root length, improving water use efficiency, and upregulating stress response genes [71,72].

Drought stress presents significant challenges to plants, triggering a complex array of molecular responses aimed at survival and adaptation. In our study of *C. oleifera*, we identified 152 additional DEGs that are crucial components of the plant’s response to drought stress. For instance, in *Helianthus annuus*, *Iris germanica*, and *Mangifera indica*, hormone-related genes were involved in the response to drought stress [73,74,75]. Hormone-related genes, such as those involved in abscisic acid (ABA)-signaling pathways, are known to mediate plant responses to drought by regulating stomatal closure and stress-responsive gene expression [76]. Upregulated SOD and POD genes can enhance the drought resistance of Triticum aestivum [77,78]. This study also found that multiple POD genes are upregulated, which is consistent with the trend of POD activity changes under drought stress. Furthermore, F-box proteins are involved in the regulation of diverse cellular processes, including responses to environmental stresses through the targeted degradation of regulatory proteins [79], and can enhance the drought tolerance of plants by regulating root structure [80] and photosynthetic performance [81]. Zinc finger proteins, characterized by their DNA-binding abilities, are crucial for the transcriptional regulation of stress-responsive genes, influencing plant growth and development under adverse conditions [82]. Cytokinin dihydrogenase (CKX) plays an important role in plant growth and development, as well as in responding to drought stress by regulating the activity of CKX enzymes [83,84]. The dual role of these DEGs as both positive and negative regulators underscores their complex involvement in *C. oleifera*’s drought adaptation strategies. By modulating various molecular pathways and physiological processes, these genes collectively contribute to the plant’s ability to withstand and recover from drought stress. Understanding their precise regulatory mechanisms will be essential for developing strategies to enhance drought tolerance in *C. oleifera* and other economically significant crops.

The findings from our qRT-PCR validation not only validate the transcriptome data, but also provide deeper insights into the molecular mechanisms underlying *C. oleifera*’s drought stress response. Future studies could further explore the specific roles of these validated genes and their regulatory networks, potentially uncovering novel targets for improving drought tolerance in *C. oleifera* and other economically important crops.

## 5. Conclusions

In this study, we conducted a comprehensive analysis of the transcriptomic response of *C. oleifera* seedlings to varying durations of drought stress. Our investigation identified a total of 21,463 DEGs, among which 18,046 genes exhibited significant changes following 15 days of drought treatment. Through GO and KEGG pathway enrichment analyses, we gained insights into the biological processes and molecular pathways activated in *C. oleifera* under drought conditions. Specifically, we focused on characterizing key gene families implicated in drought response, including protein kinases, proteases, transcription factors, and other potential regulators. These findings provide valuable insights into the genetic response of *C. oleifera* under drought stress and serve as a reference for exploring drought resistance genes, developing drought-tolerant varieties, optimizing cultivation techniques for enhanced yield, and increasing the economic value of *C. oleifera*.

However, our study has certain limitations. We did not investigate changes in hormone levels or the photosynthetic capacity of *C. oleifera* leaves under drought stress, which could have provided a more comprehensive understanding of its adaptive responses. Addressing these aspects will be pivotal in our future research endeavors to further elucidate the holistic mechanisms underlying *C. oleifera*’s response to environmental stresses. Our study contributes to the foundational knowledge necessary for advancing research on drought resilience in *C. oleifera* and other plant species, with implications for sustainable agriculture and environmental adaptation strategies.

## Figures and Tables

**Table 1:** Physiological changes of *C. oleifera* and soil volumetric moisture content under drought stress.

Treatment	POD (U g^–1^ min^–1^ FM)	CAT (U g^–1^ min^–1^ FM)	RWC (%)	Soil Volumetric Moisture Content (%)
0 d	32.31 ± 1.49 b	26.09 ± 0.66 b	60.90 ± 0.42 a	34.57 ± 1.10 a
8 d	35.00 ± 1.32 b	26.19 ± 1.05 b	55.16 ± 0.15 b	13.95 ± 0.80 b
15 d	40.65 ± 1.35 a	66.20 ± 0.43 a	43.44 ± 0.45 c	9.34 ± 0.87 c

Note: Data represent the mean ± SE (n = 3). Lowercase letters represent significant differences (*p* < 0.05) according to LSD.

**Table 2:** DEG statistics of 8 common pathways in *C. oleifera*.

Pathway	Pathway ID	0 d vs. 8 d	0 d vs. 15 d
Proteasome	ath03050	55	59
Glycolysis/Gluconeogenesis	ath00010	87	108
Galactose metabolism	ath00052	48	52
Biosynthesis of secondary metabolites	ath01110	672	1017
Protein processing in endoplasmic reticulum	ath04141	144	199
Fatty acid biosynthesis	ath00061	34	42
Peroxisome	ath04146	62	84
Fatty acid metabolism	ath01212	49	69

**Table 3:** Verification of selected differentially expressed genes using qRT-PCR.

Selected Genes	Relative Expression		
	0 d	8 d	15 d
LG01G01976 (MAPK)	0.91	1.92	1.01
LG12G00734 (Calcium-dependent protein kinase)	0.82	1.16	0.88
LG07G00954 (Serine/threonine-protein kinase)	1.14	1.43	1.30
LG02G03733 (Cysteine protease)	1.16	2.32	1.37
LG08G01571 (NAC)	1.07	1.18	1.28
LG04G00063 (ERF)	0.95	1.36	0.98
LG03G00646 (WRKY)	1.02	1.17	1.48

## Data Availability

The data can be provided by the authors upon reasonable request.

## Supplementary Materials

The following supporting information can be downloaded at: https://www.mdpi.com/article/10.3390/life14080989/s1, Table S1: Transcriptome Sequencing and Assembly of *C. oleifera*; Table S2: The top 20 GO terms of DEGs after drought treatment; Table S3: DEGs of Protein Kinases and Proteases under Drought; Table S4: DEGs of TFs under Drought; Table S5: Other Important DEGs under Drought; Table S6: Specific primers of selected genes for qRT-PCR analysis.
